# Heteropathogenic virulence and phylogeny reveal phased pathogenic metamorphosis in *Escherichia coli* O2:H6

**DOI:** 10.1002/emmm.201303133

**Published:** 2014-01-10

**Authors:** Martina Bielaszewska, Roswitha Schiller, Lydia Lammers, Andreas Bauwens, Angelika Fruth, Barbara Middendorf, M Alexander Schmidt, Phillip I Tarr, Ulrich Dobrindt, Helge Karch, Alexander Mellmann

**Affiliations:** 1 Institute of Hygiene, University of MünsterMünster, Germany; 2 Institute of Molecular Infection Biology, University of WürzburgWürzburg, Germany; 3 National Reference Center for Salmonella and Other Bacterial Enteric Pathogens, Robert Koch InstituteWernigerode, Germany; 4 NInstitute of Infectiology, Center for Molecular Biology of Inflammation (ZMBE), University of MünsterMünster, Germany; 5 Department of Pediatrics, Washington University School of MedicineSaint Louis, MO, USA

**Keywords:** heteropathogenicity, phased metamorphosis, phylogeny, Shiga toxin-producing *Escherichia coli*, uropathogenic *Escherichia coli*

## Abstract

Extraintestinal pathogenic and intestinal pathogenic (diarrheagenic) *Escherichia coli* differ phylogenetically and by virulence profiles. Classic theory teaches simple linear descent in this species, where non-pathogens acquire virulence traits and emerge as pathogens. However, diarrheagenic Shiga toxin-producing *E*.* coli* (STEC) O2:H6 not only possess and express virulence factors associated with diarrheagenic and uropathogenic *E. coli* but also cause diarrhea and urinary tract infections. These organisms are phylogenetically positioned between members of an intestinal pathogenic group (STEC) and extraintestinal pathogenic *E. coli*. STEC O2:H6 is, therefore, a ‘heteropathogen,’ and the first such hybrid virulent *E. coli* identified. The phylogeny of these *E. coli* and the repertoire of virulence traits they possess compel consideration of an alternate view of pathogen emergence, whereby one pathogroup of *E. coli* undergoes phased metamorphosis into another. By understanding the evolutionary mechanisms of bacterial pathogens, rational strategies for counteracting their detrimental effects on humans can be developed.

**Subject Categories** Microbiology, Virology & Host Pathogen Interaction

## Introduction

*Escherichia coli* are usually harmless inhabitants of the human gut. However, some members of this species have acquired specific virulence attributes that allow them to cause intestinal as well as extraintestinal diseases in humans (Kaper *et al*, 2004). One important and instructive group of intestinal pathogenic *E*. *coli* is the set of Shiga toxin (Stx)-producing *E. coli* (STEC). STEC cause diarrhea, bloody diarrhea, and, because of toxemia and not dissemination, the hemolytic uremic syndrome (HUS) (Kaper *et al*, 2004; Karch *et al*, 2005). *E. coli* O157:H7 is the most common human pathogenic STEC (Karch *et al*, 2005), but a variety of non-O157:H7 STEC serotypes have also been isolated from patients (Karch *et al*, 2005; Mellmann *et al*, 2008; Bielaszewska *et al*, 2013). These pathogens have acquired a rather stereotyped suite of virulence loci (‘parallel evolution’) (Reid *et al*, 2000; Ogura *et al*, 2009). Other well-defined intestinal *E. coli* pathogroups include enteropathogenic (EPEC), enterotoxigenic (ETEC), enteroinvasive (EIEC), enteroaggregative (EAEC), and adherent-invasive (AIEC) *E. coli* (Nataro ' Kaper, 1998; Darfeuille-Michaud, 2002; Kaper *et al*, 2004). Extraintestinal pathogenic *E. coli* (ExPEC) are classified as uropathogenic (UPEC), sepsis-associated, and meningitis-associated (MNEC) (Kaper *et al*, 2004).

*Escherichia coli* virulence and phylogeny are intertwined. Each pathogenic *E. coli* group possesses ‘signature’ repertoires of virulence genes, which enable them to colonize and injure their host (Kaper *et al*, 2004). While exceptions exist, particularly among ETEC (Turner *et al*, 2006), strains within pathogroups are often phylogenetically closely related (Achtman *et al*, 1983; Wirth *et al*, 2006). Current concepts of pathogen emergence employ linear descent scenarios, where horizontal acquisition of pathogenicity islands, bacteriophages, and plasmids by non-pathogens results in phylogenetically fixed pathogroups (Dozois ' Curtiss, 1999). Here, we determined the phylogenetic relationship of STEC O2:H6 isolated from patients with diarrhea to other intestinal and extraintestinal pathogenic *E. coli* and then characterized the O2:H6 virulence genes as well as their UPEC virulence potential. To our surprise, these analyses did not portray the linear emergence of pathogenicity by step-wise recombination events, but, instead, identified STEC O2:H6 as a ‘transitional’ pathogen in the process of morphing between pathogroups.

## Results

### Phylogeny of STEC O2:H6

**Figure 1:**
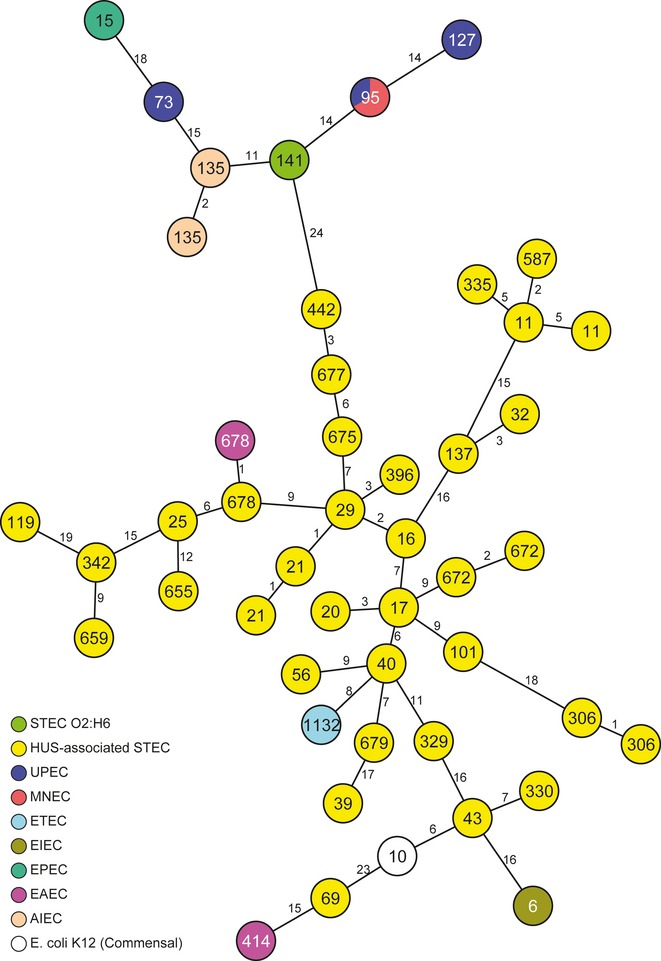
Phylogenetic relationships of STEC O2:H6 to intestinal and extraintestinal pathogenic *Escherichia coli* and to *E. coli*. K-12. Minimum spanning tree based on MLST and rMLST allelic profiles portraying the clonal relationships of STEC O2:H6 to HUS-associated STEC (HUSEC collection) (Mellmann *et al*, 2008), other intestinal pathogenic *E. coli* (EPEC, ETEC, EIEC, EAEC, AIEC), prototypic ExPEC including UPEC and MNEC isolates, and a non-pathogenic *E. coli* strain K-12 (MG1655). Isolates are described in supplementary Table S1. Each circle represents a given allelic profile (combination of MLST and rMLST loci) and is named with the MLST sequence type. The different groups of strains are distinguished by colors of the circles. The numbers on the connecting lines illustrate the number of differing alleles.

Multilocus sequence typing (MLST) (Wirth *et al*, 2006) demonstrates that 13 STEC O2:H6 strains isolated between 2000 and 2009 from epidemiologically unrelated patients with non-bloody diarrhea, whose illnesses resolved without progression to HUS and whose stools contained no other intestinal pathogens belong to sequence type (ST) 141 (supplementary Table S1). This ST is only distantly related to the STs of the non-H6 STEC O2, and is not found among STEC causing HUS (‘the HUSEC collection’) (Mellmann *et al*, 2008), intestinal pathogenic *E. coli* strains of other pathogroups (EPEC, ETEC, EIEC, EAEC, AIEC), prototypic ExPEC (UPEC and MNEC) strains, and in a non-pathogenic *E. coli* K-12 (supplementary Table S1). Intriguingly, in the minimum spanning tree based on allelic profiles of the seven MLST housekeeping genes in combination with 53 genes encoding the bacterial ribosome protein subunits (rMLST) (Jolley *et al*, 2012) (supplementary Table S2), STEC O2:H6 is positioned between the HUSEC and ExPEC (including UPEC and MNEC) strains, in the proximity of AIEC (Fig 1). In further phylogrouping (Clermont *et al*, 2000), the STEC O2:H6 isolates localize to *E. coli* Reference (ECOR) (Selander *et al*, 1987) phylogenetic group B2, as do UPEC, MNEC (Table 1) and AIEC (Dreux *et al*, 2013). In contrast, non-H6 STEC O2 and STEC in the HUSEC collection (http://www.ehec.org) belong to phylogroups A, B1, and D (Table 1).

**Table:** Virulence loci and phylogeny of STEC O2:H6 as compared to non-H6 STEC O2, HUSEC and prototypic UPEC and MNEC strains

	Presence of the locus in strain(s) (% no. of strains)^b^										
Gene or gene cluster of^a^	O2:H6 STEC	O2:H4 STEC	O2:H27 STEC	O2:H29 STEC	HUSEC STEC	536 UPEC	CFT073 UPEC	J96 UPEC	UTI89 UPEC	S88 MNEC	IHE3034 MNEC
STEC											
*stx*^c^	13 (100) (*stx*_2b_)	+ (n.k.)	+ (*stx*_2a_)	+ (*stx*_2c_)	42 (100) (*stx*_1a_, *stx*_1c_, *stx*_2a_, *stx*_2b_, *stx*_2c_, *stx*_2d_)	−	−	−	−	−	−
*saa*	13 (100)	−	−	−	6 (14.3)	−	−	−	−	−	−
ExPEC											
α*-hlyA*	10 (76.9)^d^	−	−	−	1 (2.4)	+	+	+	+	−	−
*cnf1*	10 (76.9)^d^	−	−	−	0	−	−	+	+	−	−
*vat*	13 (100)	−	−	−	0	+	+	+	+	+	+
*clb* island	13 (100)	−	−	−	0	+	+	+	+	−	+
*pap* cluster	10 (76.9)^d^	−	−	−	0	+	+	+	+	+	−
*sfa*II cluster	9 (69.2)	−	−	−	0	−	−	−	+	−	+
*hek*	10 (76.9)^d^	−	−	−	1 (2.4)	+	−	+	+	−	+
*cdiAB* cluster	13 (100)	−	−	+	6 (14.3)	+	+	+	+	−	−
*iro* cluster	12 (92.3)	−	−	−	0	+	+	+	+	+	+
*ybt* cluster	13 (100)	+	−	−	13 (31.0)	+	+	+	+	+	+
Phylogroup	B2	D	A	B1	A, B1, D	B2	B2	B2	B2	B2	B2
ST (MLST)^e^	141	405	10	515	See Fig 1	127	73	12	95	95	95

a The genes encode following proteins: *stx*, Shiga toxin; *saa*, STEC autoagglutinating adhesin; α-*hlyA*, α-hemolysin; *cnf1*, cytotoxic necrotizing factor 1; *vat*, vacuolating autotransporter toxin; *clb* island, colibactin; *pap* cluster, P fimbriae; *sfa*II cluster*,* S fimbriae, subtype SfaII; *hek*, Hek adhesin; *cdiAB*, contact-dependent inhibition phenotype; *iro* cluster and *ybt* cluster, yersiniabactin siderophore systems.

b In STEC O2:H6 (*n* = 13) and HUSEC strains (*n* = 42), the number (%) of strains positive for the locus is shown; for non-H6 O2 STEC and UPEC and MNEC strains (each *n* = 1), the presence (+) or absence (−) of the gene is indicated.

c *stx* subtypes are shown. *stx*_2_ (GenBank accession no. GU126552) subtyped as *stx*_2b_ is present in all sequenced STEC O2:H6 (supplementary Table S1). For *stx* genes in HUSEC strains see http://www.ehec.org and (Mellmann *et al*, 2008). STEC O2:H4 lost *stx* before subtyping (*stx* subtype is not known (n.k.)).

d The genes are present in the same strains.

e ST, sequence type; MLST, multilocus sequence typing.

**Figure 2:**
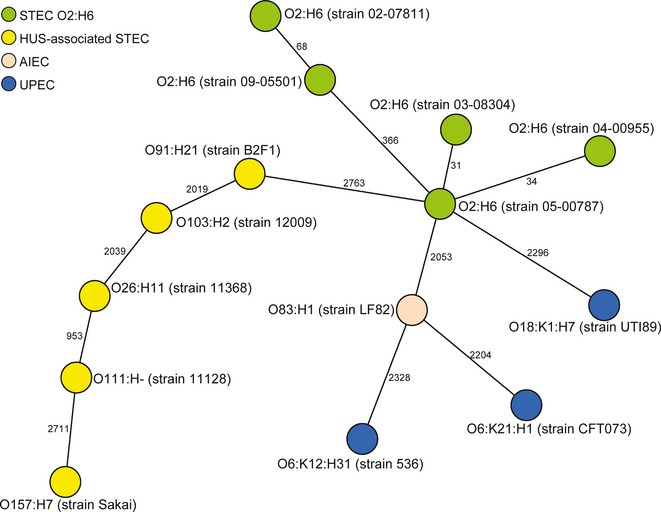
Phylogenetic relationships of STEC O2:H6 to prototypic UPEC, AIEC and most closely related and prototypic HUS-associated STEC based on whole genome sequencing. Minimum spanning tree is based on allelic profiles of 2827 genes present in all strains investigated (see supplementary Table S1). The different pathotypes are distinguished by colors of the circles and the serotypes and strain numbers (in parentheses) are given.

To more thoroughly analyze the phylogenetic relationships between STEC O2:H6, most closely related STEC (serotype O91:H21; ST442; Fig 1) and prototypic HUS-associated STEC, UPEC and AIEC, we used whole genome sequencing and a gene-by-gene analysis of in total 2827 open reading frames that were present in all 14 strains investigated (see supplementary Table S1). This analysis confirmed the intermediate positioning of STEC O2:H6 between the major HUS-associated STEC serotypes and UPEC. Interestingly, AIEC were grouped closely to UPEC (Fig 2).

### Virulence gene census of STEC O2:H6

We next sought various virulence genes of STEC, other intestinal pathogenic *E. coli*, and ExPEC in the STEC O2:H6 isolates using PCR and sequencing. STEC loci, including a *stx*_2_ allele (GenBank accession no. GU126552) that is subtyped as *stx*_2b_ encoding Stx2b (Scheutz *et al*, 2012), and *saa* (encoding STEC autoagglutinating adhesin; Saa) (Paton *et al*, 2001) were present in all STEC O2:H6 (Table 1). In contrast, we failed to find loci typical for EPEC, including genes of the locus of enterocyte effacement (LEE) (supplementary Table S3), which are in most, but not all, HUSEC (Mellmann *et al*, 2008). Also, none of the STEC O2:H6 possessed virulence factors typically found in ETEC (heat-labile and heat-stable enterotoxins), EIEC (the invasive plasmid pInv and *Shigella* enterotoxin 2), and EAEC (the EAEC heat-stable enterotoxin 1, *Shigella* enterotoxin 1, the autotransporters Pet and Pic, and the EAEC virulence plasmid) (Nataro ' Kaper, 1998; Vila *et al*, 2000; Kaper *et al*, 2004) (supplementary Table S3).

Because of their phylogenetic positioning between HUSEC and ExPEC (Fig 1), and because *E. coli* O2:H6 have been isolated from patients with urinary tract infections (Johnson *et al*, 2005), we tested the STEC O2:H6 isolates for virulence factors of ExPEC. Indeed, all STEC O2:H6 contain virulence genes typical of UPEC (Table 1). These include putative or demonstrated urovirulence loci encoding toxins (α-*hlyA*, *cnf1*, *vat*, *clb* island) (Johnson, 1991; Johnson ' Stell, 2000; Parreira ' Gyles, 2003; Johnson *et al*, 2005; Nougayrède *et al*, 2006), adhesins (*pap* cluster, *sfaII* cluster, *hek*) (Korhonen *et al*, 1982; Johnson, 1991; Hacker *et al*, 1993; Dobrindt *et al*, 2002; Johnson *et al*, 2005), the contact-dependent inhibition phenotype (*cdiAB* cluster) (Aoki *et al*, 2005), and iron acquisition systems (*iro* cluster, yersiniabactin cluster) (Johnson ' Stell, 2000; Dobrindt *et al*, 2002). Notably, these ExPEC virulence genes were rare in non-H6 STEC O2 and in strains of the HUSEC collection (Table 1).

As AIEC were also phylogenetically closely related to UPEC and to STEC O2:H6 (Figs 1 and 2), we further investigated whether STEC O2:H6 strains contain FimH belonging to the same clade as that possessed by UPEC and AIEC strains (Sepehri *et al*, 2009). FimH is the adhesin subunit of the type 1 pili that mediate adherence and play an essential role in the invasive ability of AIEC (Boudeau *et al*, 2001). Comparative sequence analysis of FimH from the sequenced STEC O2:H6 strains (supplementary Table S1) and from the UPEC (strains 536, UTI89, CFT073) and AIEC (strains LF82, LF73) reference strains showed the phylogenetic positioning of all FimH proteins of STEC O2:H6 in the S70/N78 FimH clade (supplementary Fig S1), which is typical for UPEC and AIEC (Dreux *et al*, 2013).

### Expression of STEC and UPEC virulence genes in STEC O2:H6

All STEC O2:H6 expressed Stx, as evidenced by the cytotoxicity of their supernatants to Vero cells (reciprocal titer range, 8–256; median, 32), and Saa (supplementary Fig S2).

**Figure 3:**
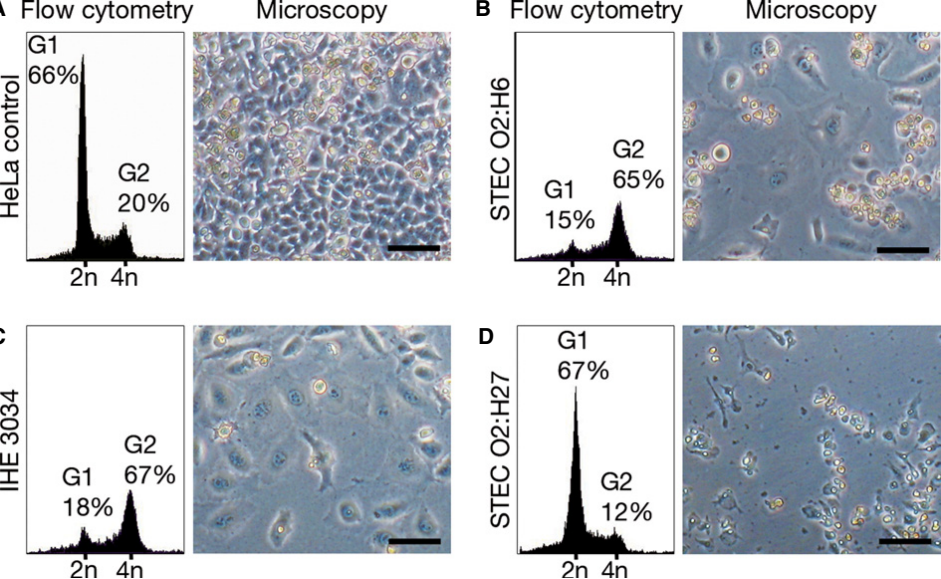
Production of colibactin by STEC O2:H6. HeLa cells were cocultured with bacteria (4 h), washed and incubated in gentamicin-supplemented medium (48 h). The DNA content was determined by flow cytometry and morphological changes were assessed microscopically. Bar = 100 μm. A Uninfected (control) cells were mostly in the G1 phase of the cell cycle (2n DNA) and retained normal morphology. B–C Cells infected with *clb*-positive STEC O2:H6 strain 05-00787 (B) or the prototypic *clb*-harboring strain IHE3034 (C) were arrested in the G2 phase (4n DNA) and converted into megalocytes. (The phenotype shown in (B) was produced by each of three *clb*-positive, α-*hlyA*-negative and *cnf1*-negative STEC O2:H6). D Cells infected with *clb*-negative Stx2-producing O2:H27 isolate displayed neither G2 arrest nor distension.

Nearly all of the identified UPEC virulence loci were also expressed by the cognate STEC O2:H6 isolates. Each of the ten STEC O2:H6 that harbored α-*hlyA* and *cnf1* (Table 1) produced α-hemolysin on blood agar and cytotoxic necrotizing factor (CNF) 1, detectable as an ∼115-kDa band in an immunoblot (supplementary Fig S3). Each of the three α-*hlyA*-negative, *cnf1*-negative and *clb-*positive STEC O2:H6 (Table 1) produced colibactin, a hybrid polyketide-peptide cyclomodulin encoded by the *clb* island (Nougayrède *et al*, 2006). Similar to prototypic colibactin-producing MNEC strain IHE3034 (Nougayrède *et al*, 2006), each STEC O2:H6 strain arrested HeLa cells in the G2 phase of the cell cycle after 48 h and distended these epithelial cells and their nuclei, converting them into megalocytes (Fig 3). In the ten remaining *clb*-positive isolates, HeLa cell lysis caused by α-hemolysin confounded our attempts to study colibactin expression. Neither G2 arrest nor cell distension were elicited by a *clb*-negative, Stx2-producing STEC O2:H27 (Fig 3), excluding a contribution of Stx to these effects.

**Figure 4:**
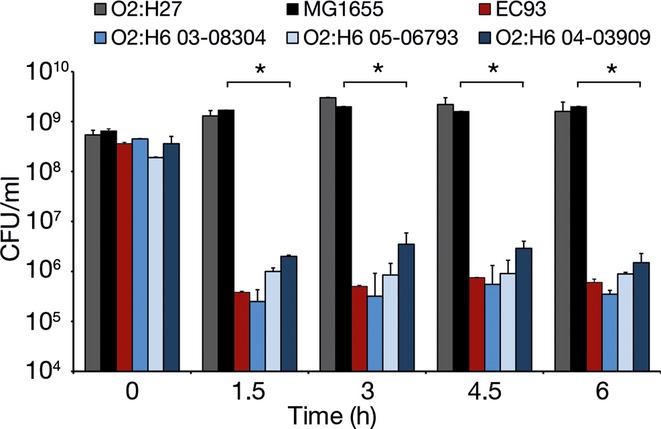
Contact-dependent growth inhibition mediated by STEC O2:H6. Ampicillin-resistant target strain MG1655/pBlueskript KS II(+) was cultured alone or in mixture with log-phase culture of each inhibitor (inhibitor-to-target ratio 50:1) including *cdiAB*-positive STEC O2:H6 strains 03-08304, 05-06793, 04-03909, prototypic *cdiAB*-harboring strain EC93, or *cdiAB*-negative STEC O2:H27. At each indicated time point, the growth of the target strain (CFU/ml) was determined by plating 10-fold culture dilutions on LB agar with ampicillin. Data represent means ± standard deviations of three independent experiments. *P <* *0.01 (unpaired Student′s *t*-test), differences between growth of the target strain alone and in coculture with each respective inhibitor.

The contact-dependent growth inhibition phenotype (Aoki *et al*, 2005), encoded by the *cdiAB* cluster, was sought in three randomly selected STEC O2:H6 (Fig 4). Each of these strains inhibited growth of the target *E. coli* MG1655/pBluescript KS II(+) strain during the 6 h observation period, as did the prototypic *cdiAB*-harboring strain EC93 (Aoki *et al*, 2005). In contrast, *cdiAB*-negative STEC O2:H27 had no inhibitory effect (Fig 4).

**Figure 5:**
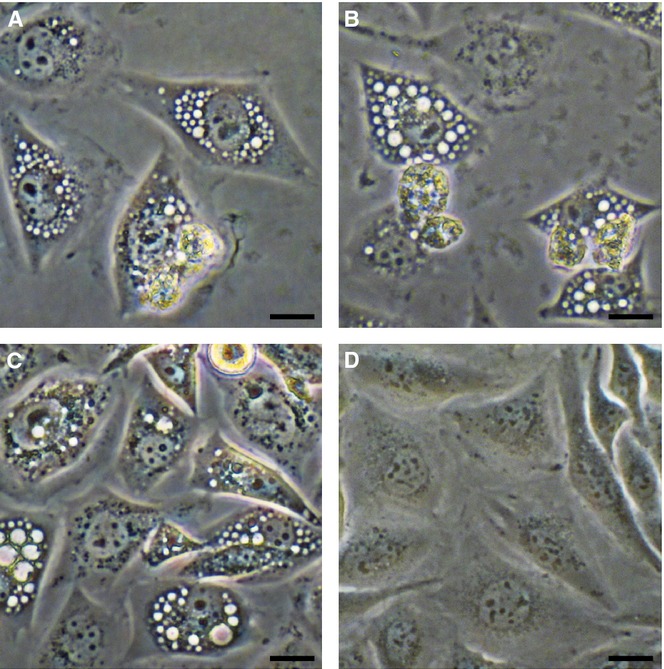
Vacuolization induced by STEC O2:H6. CHO cells were exposed to sterile culture supernatants of tested strains and presence of vacuoles was sought microscopically after 24 h. Bar = 20 μm. A–B *vat*-positive STEC O2:H6 strains 05-00787 (A) and 05-06739 (B). (Vacuolization similar to that displayed by these two strains was elicited by all STEC O2:H6 isolates). C *vat*-containing UPEC strain J96 (positive control). D Uninfected cells (negative control).

*vat*, encoding vacuolating autotransporter toxin (Vat) (Parreira ' Gyles, 2003), was expressed in all 13 *vat*-positive STEC O2:H6 as demonstrated by the ability of culture supernatants to produce vacuoles in Chinese hamster ovary (CHO) cells (Fig 5).

P and S fimbriae were expressed in eight of ten and six of nine strains, harboring these respective loci (Table 1), as demonstrated by the ability of the bacteria to agglutinate human and bovine erythrocytes, respectively, in the presence of mannose (Blumer *et al*, 2005). Thus, the UPEC virulence loci found in STEC O2:H6 are largely functional across the collection of strains that we studied.

### Analysis of urovirulence of STEC O2:H6

**Figure 6:**
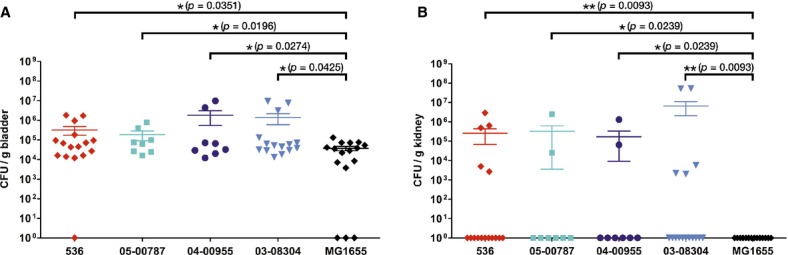
Urovirulence of STEC O2:H6 strains. Bladder (A) and kidney (B) colonization levels were determined 72 h after transurethral inoculation of mice with UPEC strain 536 (positive control), the STEC O2:H6 strains 05-00787, 04-00955, and 03-08304, or non-pathogenic *Escherichia coli* K-12 strain MG1655 (negative control). Horizontal bars represent the mean CFU number of each strain per gram of tissue; the whiskers display the respective standard error of the mean. Significant differences in the bacterial organ load compared to the negative control are indicated by asterisks.

The ability of randomly selected STEC O2:H6 isolates to cause urinary tract infection (UTI) was tested in an experimental murine model of ascending UTI. The bacterial numbers in the bladder and the kidneys were determined 72 h after infection (Fig 6). The prototypic UPEC strain 536 and STEC O2:H6 strain 05-00787 were recovered in nearly equal numbers from the bladder tissue (1 × 10^5^–1 × 10^6^ colony-forming units (CFU)/g bladder) (Fig 6A). Bladder colonization by STEC O2:H6 strains 04-00955 and 03-08304 resulted in 10-fold higher organ loads relative to UPEC 536. In all four cases, bacterial concentrations in the bladder tissue were significantly higher than after infection with non-pathogenic *E. coli* K-12 strain MG1655 (Fig 6A). All three STEC O2:H6 strains also colonized the kidneys as efficiently as UPEC strain 536 (Fig 6B). In contrast, strain MG1655 was unable to ascend to the kidneys (Fig 6B). Consequently, the potential of STEC O2:H6 strains to cause UTI in this model is comparable to that of classic UPEC strain 536.

## Discussion

The clinical significance of hybrid pathogens was clearly demonstrated by the deadly 2011 outbreak caused by *E. coli* O104:H4 (Bielaszewska *et al*, 2011; Frank *et al*, 2011; Karch *et al*, 2012). The outbreak started in Germany in May 2011 and subsequently spread to other European countries and North America, affecting in total nearly 4000 persons of which more than 900 developed HUS and 54 died (Karch *et al*, 2012). The outbreak strain uniquely combined virulence genes typical for STEC and EAEC and expressed the phenotypes that define these pathogroups including Stx2 production and aggregative adherence to intestinal epithelial cells (Bielaszewska *et al*, 2011; Brzuszkiewicz *et al*, 2011; Mellmann *et al*, 2011; Rasko *et al*, 2011), the features that likely increased its virulence. This outbreak, therefore, tragically illustrated that blended virulence profiles in enteric pathogens introduced into susceptible populations can have serious public health consequences (Karch *et al*, 2012). Moreover, this outbreak highlighted the lack of our knowledge of the basic principles of evolutionary trends of new pathogens as demonstrated by the fact that the origin of the *E. coli* O104:H4 hybrid and its evolutionary history remain obscure.

In this context, our identification of another hybrid pathogen, STEC O2:H6 in this study, and gaining insight into its evolutionary role is of particular importance, because we can determine the phylogenetic coordinates of its evolution. Also, STEC O2:H6 is the first hybrid pathogen to demonstrate pluripotential pathogenicity in intestinal and extraintestinal milieus, as predicted by its virulence repertoire. Specifically, STEC O2:H6 occupies an evolutionary and pathogenic interface between intestinal and extraintestinal pathogenic *E. coli*, and several lines of evidence corroborate this assignment: sequence typing, virulence genotyping and phenotyping, and the clinical potential. First, MLST/rMLST, employing sequences of a sample of housekeeping and ribomosal genes scattered around a chromosomal backbone, agnostically placed these isolates between HUSEC and ExPEC (Fig 1). Also whole genome sequencing of STEC O2:H6, closely related HUS-associated STEC, and prototypic HUSEC, AIEC and UPEC strains positioned STEC O2:H6 separately from the other pathotypes in an intermediate position (Fig 2). Next, STEC O2:H6 contain UPEC and HUSEC virulence genotypes and phenotypes, using ascertainments independent of sequence typing, a finding that appears highly non-random. While it is possible that chance acquisition of a gene encoding Stx via phage transduction into either a commensal *E. coli* or an ExPEC could have resulted in STEC O2:H6, our data argue in favor of true UPEC/HUSEC heteropathogenicity. Specifically, the phylogeny places STEC O2:H6 at quite a distance from commensal *E. coli*, and at moderate distances from ExPEC (including UPEC) and from HUSEC (Fig 1). Hence, the STEC O2:H6 are incarnated as a phylogenetically recognizable group, and one that differs from UPEC (Figs 1 and 2). Also, an additional virulence gene found in a subset of HUSEC, *saa*, encoded independently on a plasmid (Paton *et al*, 2001) (unlike *stx* which is encoded on a bacteriophage that integrates into the chromosome) was present in all STEC O2:H6, in addition to the many UPEC virulence loci. This simultaneous convergence on UPEC and STEC phylogeny and virulence profiles is most simply explained by a phased transition from one group of pathogens to the other. This is also corroborated by the whole genome sequence data, where STEC O2:H6 strains represent a separate lineage (Fig 2) indicating that the core genome has co-evolved with the virulence attributes. Finally, the heteropathogenic clinical potential of STEC O2:H6 is demonstrated by their ability to cause diarrhea in the human host and experimental UTI in mice. The evolution of STEC O2:H6 via a phased pathotype transition is overall different from that of the *E. coli* O104:H4 hybrid, which combines an EAEC genomic background with the presence of *stx* characteristic of STEC. However, both evolutionary models of this pathogen (Brzuszkiewicz *et al*, 2011; Mellmann *et al*, 2011; Rasko *et al*, 2011) suggest a classic linear evolution from a progenitor with reduced intestinal virulence via loss and/or acquisition of various mobile elements such as bacteriophages, genomic islands or plasmids (Brzuszkiewicz *et al*, 2011; Mellmann *et al*, 2011; Rasko *et al*, 2011). AIEC are, similar to STEC O2:H6, phylogenetically located between ExPEC (including UPEC) and intestinal pathogenic *E. coli* (Fig 1). Our in-depth analysis based on the whole genome sequencing and core genome analysis corroborated this fact and positioned AIEC even closer to UPEC (Fig 2). These data, together with the recently published genome sequence of the prototypic AIEC isolate LF82 (Miquel *et al*, 2010), demonstrate the genetic hybrid character of AIEC. However, as AIEC strains have only been associated with Crohn's disease (Darfeuille-Michaud *et al*, 1998; Darfeuille-Michaud, 2002) and not detected in human extraintestinal diseases, they cannot be considered, in contrast to STEC O2:H6, as heteropathogens from the clinical standpoint.

The heteropathogenicity of *E. coli* O2:H6 has multiple implications for our conceptualization of bacterial evolution and pathogen emergence. First, and most significantly, the ‘missing link’ phylogeny and virulence traits of STEC O2:H6 suggest that pathogenic *E. coli* emerge not only by the simple linear acquisition of virulence loci by non-pathogenic *E. coli* strains. We speculate, based on our data, that one pathogroup gradually exchanges one suite of virulence loci for those of another pathogroup, as its core genome transitions simultaneously and in the same direction in a process of ‘phased metamorphosis’. This emergence model differs considerably from a linear ‘pathogenic stem cell’ descent scenario because metamorphosis implies that a pathogenic *E. coli* in a given venue and phylogeny retains alternative virulence options. Certainly, the step-wise scenario whereby non-virulent (or less virulent) progenitors of pathogens acquire virulence genes resulting in pathogen emergence (Dozois ' Curtiss, 1999) is appropriate for tightly circumscribed groups of pathogens, such as the enterohemorrhagic *E. coli* (EHEC) 1 clade (Leopold *et al*, 2009) or the recent *E. coli* O104:H4 outbreak strain, where, most plausibly, an EAEC acquired a Stx2-encoding bacteriophage (Bielaszewska *et al*, 2011; Brzuszkiewicz *et al*, 2011; Mellmann *et al*, 2011; Rasko *et al*, 2011). We wish to note that the latter pathogen, while possessing blended genotypes and phenotypes of EAEC and STEC, has a largely linear descent method of evolution, because it is phylogenetically closely related to an EAEC prototype strain 55989 (Brzuszkiewicz *et al*, 2011; Mellmann *et al*, 2011; Rasko *et al*, 2011). However, linear models might not apply across the broader *E. coli* genospecies, where evolution and pathogen emergence might be more trabeculated. Our data are compatible with a recent description of the complex and highly interwoven evolutionary history of *E. coli* (Touchon *et al*, 2009), including the flow of specific ‘highways’ of gene exchange (Leopold *et al*, 2011).

Our data actually validate the concept that virulence is ordained by phylogeny as well as by genes encoding specific effector molecules (Whittam *et al*, 1993). Specifically, the phylogenetic positioning of the STEC O2:H6 isolates between STEC and ExPEC (including UPEC) (Figs 1 and 2) and their possession of virulence loci from both STEC and UPEC (Table 1), reflect the co-evolution of the core chromosome with the accrual of virulence traits of the two closest pathogroups. Notably, loci specific for other pathogroups, such as those common to EPEC, ETEC, EIEC, or EAEC, are not present.

Shiga toxin-producing *Escherichia coli* are rare among urinary strain set collections. Johnson *et al* (Johnson *et al*, 2002) screened 597 UPEC isolates, and found no STEC, even though presumably non-toxigenic *E. coli* O2:H6 might have been in this collection. Indeed, *E. coli* O2:H6 accounted for 2.9% of UPEC isolated by these investigators in another study (Johnson *et al*, 2005). STEC O2:H6 is also rare in fecal samples from patients with diarrhea (Piérard *et al*, 1990) and absent from the HUSEC collection (Mellmann *et al*, 2008). The paucity of these organisms in large strain collections prompts us to speculate that possession of traits of intestinal and urinary pathogroups might reduce the ability of an organism to cause disease in either venue. Nevertheless, the heteropathogenic potential of these strains is substantiated by their isolation as the only pathogens from stools of epidemiologically unrelated patients with diarrhea in this study, and their identification as diarrheagenic in other studies (Piérard *et al*, 1990), and their ability to cause UTI in an animal model.

The heteropathogenic nature of STEC O2:H6 warrants additional comments. The transitional nature of phased metamorphosis differs from parallel and convergent evolution. Specifically, this model portrays pathogens evolving along a continuum, with the backbone changing in synchrony with virulence loci. Our data clearly recommend combining backbone phylogeny assignment by MLST/rMLST and whole genome sequencing with a broad spectrum virulence profiling to discern metamorphosis between *E. coli* pathogroups. While backbone analysis (i.e. the combination of MLST, rMLST and whole genome sequencing in this case) accomplishes phylogenetic positioning, its combination with virulence profiles provides a more textured picture of evolution, and enabled us to propose this alternate mechanism of pathogen emergence. Application of such orthogonal assessments to additional isolates in other serotypes should determine the extent to which other heteropathogenic *E. coli* are found among the *E. coli* species, an association that might be obscured by microbiologists′ focusing only on diagnostically and pathogenetically relevant virulence factors.

In summary, STEC O2:H6 is an extant ‘way station’ between major groups of pathogenic *E. coli*. It affords a unique opportunity to study pathogen emergence via pathogroup conversion, and introduces phased metamorphosis as a new evolutionary concept. Broader surveys combining backbone phylogeny and systematic virulence gene enumeration will be needed to determine if this form of pathogen emergence via transition is a generalized process.

## Materials and Methods

### Bacterial strains

The STEC O2, other intestinal pathogenic *E. coli*, and ExPEC strains used in this study are listed in supplementary Table S1. Except for an STEC O2:H29 (Tasara *et al*, 2008) (gift of R. Stephan, University of Zürich, Zürich, Switzerland), all the other STEC O2 strains originated in Germany and represent all STEC of this serogroup recovered in the European authors′ laboratories. HUSEC strains (Mellmann *et al*, 2008) and their characteristics are available at http://www.ehec.org. The MLST data and the phylogenetic groups of the EPEC, ETEC, EIEC, EAEC, AIEC, UPEC and MNEC strains, and *E. coli* K-12 strain MG1655 were derived from the published genome sequences at http://www.ncbi.nlm.nih.gov. *E. coli* strain EC93 used as a control in growth inhibition experiments has been previously described (Aoki *et al*, 2005) and was a gift of D. A. Low (University of California-Santa Barbara, Santa Barbara, CA, USA).

### MLST, rMLST, PCR phylogrouping and whole genome sequencing

Internal fragments of seven housekeeping genes were sequenced (Wirth *et al*, 2006) and STs were assigned according to the *E. coli* MLST website (http://mlst.ucc.ie/mlst/dbs/Ecoli). Similarly, sequences of the 53 rMLST loci were determined, alleles were assigned in accordance to the rMLST database (Jolley *et al*, 2012) and listed in supplementary Table S2. The minimum spanning tree based on the MLST and rMLST allelic profiles (in total 60 loci, ca. 24.6 kb) was generated using the SeqSphere software version 0.9 beta (Ridom GmbH, Münster, Germany). Classification into ECOR phylogenetic groups A, B1, B2, and D was performed as described (Clermont *et al*, 2000). For whole genome shotgun sequencing of selected O2:H6 strains (see supplementary Table S1), sequencing libraries were prepared using the Nextera XT chemistry (Illumina Inc., San Diego, CA, USA) for either a 100 bp or a 250 bp paired-end sequencing run on an Illumina HiScanSQ or MiSeq sequencer in accordance to the manufacturer′s recommendations (Illumina). After quality trimming using the default parameters of the CLC Genomic Workbench software (CLC bio, Arhus, Denmark) the sequencing reads were assembled using the CLC Genomic Workbench *de novo* assembler (CLC bio). Gene sequences for subsequent analyses were extracted from contigs using the Ridom Seqsphere software version 0.9 beta (Ridom GmbH). For the gene-by-gene core genome analysis as described (Mellmann *et al*, 2011), we included all genes present in all strains analyzed (see supplementary Table S1). The whole genome sequence reads have been deposited at ENA SRA (study accession no. PRJEB4756).

### FimH sequencing

FimH protein sequences were derived from the whole genome sequences and translated using the Ridom Seqsphere software version 0.9 beta (Ridom GmbH). The Neighbor-joining tree of the FimH sequences was created using the MEGA software (Tamura *et al*, 2011). For comparison, the published sequences of AIEC/UPEC typical FimH alleles (Dreux *et al*, 2013) were included.

### Genotypic characterization

We used published PCR methodologies to find evidence of putative virulence genes associated with STEC, such as those encoding toxins (*stx*, EHEC-*hlyA*, *cdt*-III, *cdt*-V and *subAB* operons), serine proteases (*espP*, *espI*), adhesins (*eae*, *saa*, *lpfA*_O26_, *lpfA*_O113_, *lpfA*_O157-OI141_, *lpfA*_O157-OI154_, *efa1*, *sfpA*), LEE-encoded type III secretion system (*escV*) and secreted proteins (*espF*, *map*, *espG*) (Friedrich *et al*, 2003; Gauthier *et al*, 2003; Bielaszewska *et al*, 2004, 2005b; Dahan *et al*, 2004; Mairena *et al*, 2004; Paton *et al*, 2004; Toma *et al*, 2004; Brockmeyer *et al*, 2007), and with UPEC including α-*hlyA, cnf1*, *cdt*-I and *cdt*-IV operons, *vat*, *sat*, *pap* cluster (*papACEFGH*), *sfaA*I, *sfa*II cluster (*sfaAGSHII*), *focA*, *focG, sfrA*, and *hek* (Blum *et al*, 1995; Johnson ' Stell, 2000; Dobrindt *et al*, 2001, 2002; Bielaszewska *et al*, 2004; Ewers *et al*, 2005), as well as genes encoding iron acquisition systems such as *iro* cluster (*iroNEDCB*), and the yersiniabactin cluster (*ybtS*, *ybtQ*, *ybtA*, *irp2*, *irp1*, *ybtU*, *ybtT*, *ybtE*, *fyuA*) (Karch *et al*, 1999; Sorsa *et al*, 2003). The yersiniabactin cluster is characteristic of UPEC and MNEC (Johnson ' Stell, 2000; Dobrindt *et al*, 2002; Sorsa *et al*, 2003) but has also been found in a subset of STEC (Karch *et al*, 1999). Moreover, the presence of the *cdiAB* cluster and the *clb* island was sought using PCRs listed in supplementary Table S4; the specificity of amplicons from these PCRs introduced in this study was confirmed by sequence analysis using Sanger sequencing. In addition, STEC O2:H6 were PCR-tested for virulence loci typical for other intestinal pathogenic *E. coli* including ETEC (*elt* and *estI* encoding heat-labile and heat-stable enterotoxin, respectively), EIEC (*ial*, a marker for the virulence plasmid pInv, and *sen* encoding a homologue of *Shigella* enterotoxin 2), and EAEC (*set1, astA, pic* and *pet* encoding *Shigella* enterotoxin 1, EAEC heat-stable enterotoxin 1, autotransporters Pic and Pet, respectively, and *aatA*, a marker for the EAEC virulence plasmid) (Nataro ' Kaper, 1998; Vila *et al*, 2000).

### Sequence analysis of *stx* genes

*stx* genes of six randomly selected STEC O2:H6 strains (supplementary Table S1) were amplified and Sanger sequenced; resulting sequences were analyzed using the SeqSphere software version 0.9 beta (Ridom GmbH) and homologies were sought in GenBank (http://www.ncbi.nlm.nih.gov/BLAST). The *stx*_2_ sequence from strain 03-08304 (representative of those present in all six sequenced strains) (*stx*_2b_ subtype) was deposited in GenBank (accession no. GU126552).

### Phenotype determinations

Stx titers were determined by Vero cell cytotoxicity (Bielaszewska *et al*, 2006), and production of α-hemolysin was determined on Columbia blood agar (Heipha, Heidelberg, Germany) after overnight incubation. Production of Saa and CNF1 was assayed by immunoblot (Paton *et al*, 2001). Briefly, lysates of overnight Luria-Bertani (LB) broth cultures were subjected to sodium dodecyl sulfate polyacrylamide gel electrophoresis (SDS-PAGE), separated proteins were transferred to a membrane (Immobilon P, Roth, Karlsruhe, Germany) and probed with polyclonal mouse anti-Saa antibody (Paton *et al*, 2001) (a gift from J. C. Paton, Adelaide University, Adelaide, Australia) or monoclonal mouse antibody against CNF1 + CNF2 (clone JC4) (Abcam, Cambridge, UK). Bound antibodies were detected using alkaline phosphatase-conjugated goat anti-mouse IgG (Dianova, Hamburg, Germany).

Colibactin expression was tested (Nougayrède *et al*, 2006) using HeLa cells (ATCC CCL-2) maintained in Eagle minimum essential medium supplemented with 10% fetal calf serum (FCS), 2 mM l-glutamine, and 1% non-essential amino acids (Cambrex Bioscience, Verviers, Belgium). For cell cycle analysis, the cells were cultured in 12-well plates seeded with 7.5 × 10^4^ cells per well. Semiconfluent monolayers were infected with overnight LB broth cultures of tested isolates diluted in interaction medium (culture medium with 5% FCS) to a multiplicity of infection (number of bacteria per cell) ∼100:1, and cocultured for 4 h (37°C, 5% CO_2_). Cells were then extensively washed and incubated in full fresh medium supplemented with gentamicin (200 μg/ml) for 48 h. After harvesting, the cells were stained with propidium iodide-containing Nicoletti buffer (Bielaszewska *et al*, 2005a) and the DNA content of the nuclei was analyzed by flow cytometry on FACScalibur (Becton Dickinson, Heidelberg, Germany) as described previously (Bielaszewska *et al*, 2005a). For the cell distension assay, HeLa cells (3 × 10^4^ per well) were seeded into 24-well microtiter plates. Semiconfluent monolayers were exposed for 4 h to overnight bacterial cultures as described above, the cells were then washed, and incubated in full medium with gentamicin for 48 h; morphology of native cells was examined using a light microscope (Axiovert 40; Zeiss, Jena, Germany).

Contact-dependent growth inhibition was determined (Aoki *et al*, 2005) using three randomly selected *cdiAB*-positive STEC O2:H6 strains and an *cdiAB*-negative STEC O2:H27 strain as inhibitors and *E. coli* K-12 strain MG1655 transformed with pBluescript KS II(+) (Stratagene, La Jolla, CA, USA) encoding ampicillin resistance as a target strain. Briefly, a log-phase LB broth culture of each inhibitor strain was mixed (ratio 50:1) with a stationary culture of *E. coli* MG1655/pBluescript KS II(+) and cocultured (37°C, 225 rpm) for 1.5, 3, 4.5, and 6 h. At each time point, growth of the target strain incubated with each inhibitor and alone (growth control) was determined (CFU/ml) by plating 10-fold dilutions of the cultures on LB agar with ampicillin (100 μg/ml) (to which all inhibiting bacteria were susceptible).

To determine if the isolates expressed Vat, CHO-K1 cells (ACC 110; German collection of microorganisms and cell cultures, Braunschweig, Germany) were seeded in 24-well plates (4 × 10^3^ cells per well) in Ham′s F12 medium with 10% of FCS (Cambrex). The cells were incubated with sterile overnight culture supernatants of the strains for 24 h (37°C, 5% CO_2_) and vacuoles were sought by microscopy (Axiovert 40).

Expression of P- and S-fimbriae was tested using mannose-resistant (1% D-mannose; Roth) slide agglutination of 5% suspensions of defibrinated human and bovine erythrocytes (Elocin Laboratory, München, Germany), respectively (Blumer *et al*, 2005).

### Experimental murine model of ascending UTI

Mice experiments were performed according to the guidelines for the Care and Use of Laboratory Animals in compliance with German regulations (Tierschutzgesetz). Permission for this study was provided by the regional government (AZ 55.2-2531.01-53/09). Mice (C57BL/6; female, 6–7 weeks old) were obtained from Charles River Laboratories (Sulzfeld, Germany) and kept under specific pathogen-free conditions. For murine infection, the bacterial strains were grown overnight under aerated conditions in LB medium, harvested by centrifugation and resuspended in sterile saline to a concentration of 1 × 10^10^ to 1 × 10^11^ CFU/ml. Groups of 8–15 C57BL/6 mice were transurethrally inoculated with 5 × 10^8^–5 × 10^9^ CFU of strain 536 (UPEC, positive control), STEC O2:H6 strains 05-00787, 04-00955, or 03-08304, or non-pathogenic *E. coli* K-12 strain MG1655 (negative control) as previously described (Hagberg *et al*, 1983). In each case, animals were sacrificed 72 h after infection. The bladder and kidneys were removed under sterile conditions, rinsed extensively with 0.9% NaCl and homogenized mechanically in 1 ml of 0.025% Triton-X 100, 0.9% NaCl. The number of bacteria was quantified by scoring CFU after overnight culture at 37°C on LB agar plates. The results were expressed as CFU/g of tissue. Statistically significant differences in colonization levels (*P *<* *0.05) were assessed using Graphpad Prism 5 software. If the bladder challenge data were normally distributed, they were analyzed using an unpaired one-tailed *t*-test. Kidney infections were analyzed using the unpaired, non-parametric one-tailed Mann-Whitney test.

## Author contributions

HK, MB, AM and PIT designed experiments; MB, AM, LL, AB, AF, RS and BM performed experiments; MB, AM, HK, MAS, UD and PIT analyzed data; and AM, RS, MB, HK, UD and PIT wrote the manuscript.

**The paper explained**

**Problem**

Classic evolutionary theory teaches that pathogens arise from non-pathogens by horizontal acquisition of virulence genes. This theory implies a unidirectional path to virulence, and a non-pathogen to pathogen gradient. Hybrid pathogens that combine virulence traits of different pathogroups can cause severe diseases in humans but their evolutionary history is poorly understood. Specifically, we do not know if they are ‘weaponized’ by the step-wise acquisition of virulence loci, or if pathogens transition from one pathogroup to another by multi-locus emergence.

**Results**

Shiga toxin-producing *E*.* coli* (STEC) O2:H6 are phylogenetically positioned between intestinal pathogenic STEC and uropathogenic *E. coli* (UPEC). They possess and express virulence factors associated with both STEC and UPEC. This hybrid causes both diarrhea and urinary tract infection. STEC O2:H6 are thus heteropathogens that occupy an evolutionary and pathogenic interface between intestinal and extraintestinal pathogenic *E. coli*. They are in a transitional evolutionary and virulence state.

**Impact**

The phylogeny and virulence potential of STEC O2:H6 compels us to propose a novel evolutionary concept whereby one pathogroup of *E. coli* undergoes phased metamorphosis into another. Improved understanding of evolutionary mechanisms of human pathogens could inspire novel strategies to predict pathogen emergence, and counteract their detrimental effects on human hosts.

## Data deposition

The *stx* sequence reported in this paper has been deposited in GenBank (accession no. GU126552) and the whole genome sequence reads have been deposited at ENA SRA (study accession no. PRJEB4756).
