# Supplementary material for: Heteropathogenic virulence and phylogeny reveal phased pathogenic metamorphosis in Escherichia coli O2:H6
Source: EMBO Mol Med. 2014 Jan 10;6(3):347–57. doi: 10.1002/emmm.201303133 (PMC3958309; doi:10.1002/emmm.201303133)
Supplement: Supplementary file 1 [file emmm0006-0347-sd1.pdf]

Manuscript EMM-2013-03133

Heteropathogenic virulence and phylogeny reveal phased pathogenic metamorphosis in *Escherichia coli* O2:H6

Martina Bielaszewska, Roswitha Schiller, Lydia Lammers, Andreas Bauwens, Angelika Fruth, Barbara Middendorf, M. Alexander Schmidt, Phillip I. Tarr, Ulrich Dobrindt, Helge Karch, Alexander Mellmann

Corresponding author: Alexander Mellmann, University Hospital Muenster

Editor: Céline Carret

Review timeline:

Submission date:	06 June 2013
Editorial Decision:	28 June 2013
Revision received:	15 October 2013
Editorial Decision:	04 November 2013
Revision received:	08 November 2013
Accepted:	11 November 2013

Transaction Report:

(Note: With the exception of the correction of typographical or spelling errors that could be a source of ambiguity, letters and reports are not edited. The original formatting of letters and referee reports may not be reflected in this compilation.)

1st Editorial Decision 28 June 2013

Thank you for the submission of your manuscript to EMBO Molecular Medicine. We have now heard back from the two referees whom we asked to evaluate your manuscript. Although they both find the study to be of potential interest, they raise a few concerns that will need to be addressed in a revised version of your manuscript.

Both referees recommend strengthening the findings by additional comparative analyses and Ref2 also suggests sequencing some of the ST141 isolates to provide a more comprehensive bioinformatics/evolution analysis.

Given the potential interest of your study, we would be willing to consider a revised manuscript with the understanding that the reviewers' concerns must be fully addressed, with additional experiments where appropriate.

Please note that it is EMBO Molecular Medicine policy to allow a single round of revision in order to avoid the delayed publication of research findings. Consequently, acceptance or rejection of the manuscript will depend on the completeness of your responses included in the next version of the manuscript.

EMBO Molecular Medicine has a "scooping protection" policy, whereby similar findings that are published by others during review or revision are not a criterion for rejection. Should you decide to submit a revised version, I do ask that you get in touch after three months if you have not completed it, to update us on the status.

Please also contact us as soon as possible if similar work is published elsewhere. If other work is published we may not be able to extend the revision period beyond three months.

I look forward to receiving your revised manuscript.

***** Reviewer's comments *****

Referee #1 (Remarks):

The manuscript submitted by Bielaszewska and colleagues describes Escherichia coli serotype O2:H6 as an evolutionary intermediate (Shiga toxin-2b positive, intimin negative, alpha-hemolysin positive, P pilus positive) between Shiga toxin-producing E. coli (STEC) and virulent E. coli strains causing extra-intestinal infections.

GENERAL COMMENTS:
1. Adherent-invasive E. coli (AIEC, serotype O83:H1) isolated from the terminal ileum of patients with Crohn's disease also appear to be a "heteropathogen" with some features of a fecal commensal, others of a enteric pathogen, and still others of uropathogenic E. coli (UPEC).
2. A comparison with the intermediate evolution of E. coli O104:H4 (EMBO Mol Med 2012;4:841-848) should be provided.

SPECIFIC COMMENTS:
1. Title, page 1: the focus on a study of serotype O2:H6 should be included.
2. Abstract, page 2: a huge emerging literature on AIEC argues against O2:H6 being "the first such virulent E. coli identified".
3. Results, page 5, paragraph 1: additional information about the 13 O2:H6 isolates would be of interest: how many patients had bloody diarrhea? Did any patients develop hemolytic-uremic syndrome. Were other known enteric pathogens isolated?
4. Results, page 6, paragraph 2: it should be determined whether the O2:H6 strains contain type 1 pilus genes typical of UPEC and AIEC (Inflammatory Bowel Disease 2009;11: 737-745).
5. Results, page 8: is the urovirulence of O2:H6 specific? It would be of great interest to know whether O104:H4 and O83:H1 strains have comparable effects in murine models of cystitis and pylenophritis. It would also be relevant to determine whether O2:H6 isolates induce enteric disease in a relevant animal model (Nat Commun 2013; June10;4:1957).
6. Discussion, page 11, paragraph 2: phylogenetic positioning between STEC and UPEC of serotypes other than O2:H6 (eg. O83:H1) must be acknowledged and then considered in some detail.

Referee #2 (Comments on Novelty/Model System):

I basically believe that this is an interesting study on pathogen evolution, but I think they have to push the genomic analyses much further. Thus, for a paper in EMBO Molecular Medicine I think they need to make whole genome analyses of some ST141 isolates and make state of the art bioinformatic comparisons to their closest relatives among UPEC and HUSEC isolates.

Referee #2 (Remarks):

In this paper by Bielaszewska et al a set of diarrheangenic Shiga toxin producing E. coli (STEC) 02:H6 were shown to possess virulence attributes of STEC as well as uropathogenic E. coli. Phylogenetically these isolates were positioned between UPEC and HUSEC. The authors interpret their findings such that one pathogroup of E. coli may undergo phased metamorphosis from one pathogroup to another. Their findings that the core genome changes together with the virulence attributes is an argument for that virulence do not simply evolve by linear acquisition of virulence genes into a non-pathogenic background. However, the suggestion that the studied STEC ST141 isolates represent old transient hybrid pathogens that next over time may evolve into either UPEC or HUSEC is not fully convincing and difficult to distinguish from a model where these STEC isolates

are genetic end products of more recent events involving one or more large horizontal gene transfers between UPEC and HUSEC isolates. To strenghten the idea put forward by the authors I would recommend them to fully sequence some of the ST141 isolates and do the same for UPEC and HUSEC isolates that are the most related to ST141. If ST141 represents a very old lineage where the core genome has co-evolved with the virulence attributes it would look different from a more recently evolved hybrid from already existing UPEC and HUSEC genomes.
Apart from this major comment I find this paper interesting and well written.

1st Revision - authors' response 15 October 2013

Referee #1 (Remarks):

The manuscript submitted by Bielaszewska and colleagues describes Escherichia coli serotype O2:H6 as an evolutionary intermediate (Shiga toxin-2b positive, intimin negative, alpha-hemolysin positive, P pilus positive) between Shiga toxin-producing E. coli (STEC) and virulent E. coli strains causing extra-intestinal infections.

GENERAL COMMENTS:

1. Adherent-invasive E. coli (AIEC, serotype O83:H1) isolated from the terminal ileum of patients with Crohn's disease also appear to be a "heteropathogen" with some features of a fecal commensal, others of a enteric pathogen, and still others of uropathogenic E. coli (UPEC).

Answer to 1: We have included in the revised version of our manuscript genotypical data (MLST and rMLST) of two AIEC strains that were published (strains LF82 and NRG_857C, see revised Figure 1). Indeed, we were fascinated to note that both strains were located not only in the proximity to the STEC O2:H6 strains (ST141) on which our manuscript is built, but also between uropathogenic and intestinal pathogenic *E. coli* including HUS-associated STEC, ETEC, EAEC and EIEC. We now included this information in the Results section of our revised manuscript (page 5, lines 14-15) and in Figure 1. The close relationship of AIEC to STEC O2:H6 and UPEC was also confirmed by whole genome sequencing as shown in Figure 2 of the revised manuscript and mentioned in the Results section (page 5, lines 20-24, page 6, lines 1-2) and Discussion (page 11, line 26 and page 12, lines 1-5). However, despite this hybrid phylogeny, we hesitate to designate AIEC as heteropathogens without an extraintestinal pathogenic role for these organisms, beyond their association with Crohn's disease. This rationale is discussed in the revised manuscript (page 12, lines 5-8). If future data demonstrate a role for AIEC in an extra-intestinal disease, they should be easily re-classified at that time.

2. A comparison with the intermediate evolution of E. coli O104:H4 (EMBO Mol Med 2012;4:841-848) should be provided.

Answer to 2: As suggested, we have included a section in the Discussion of the revised manuscript, where we provide a comparison of the evolution of STEC O2:H6 proposed from our data with the evolutionary models of the hybrid E. coli O104:H4 outbreak strain (page 11, lines 19-26).

SPECIFIC COMMENTS:

1. Title, page 1: the focus on a study of serotype O2:H6 should be included.

Answer to 1: We have included in the revised version the serotype O2:H6 in the title as suggested by referee #1.

2. Abstract, page 2: a huge emerging literature on AIEC argues against O2:H6 being "the first such virulent E. coli identified".

Answer to 2: We agree that AEIC are closely related based on whole genome sequence analysis to ExPEC and also share virulence determinants with these strains. This might qualify them as a hybrid pathogen (please see also our Answer to general comment 1). However, AIEC, like STEC O104:H4, have only a single disease phenotype in the patient, specifically associations with Crohn's disease and diarrhea-positive HUS, respectively. In contrast, STEC O2:H6 described here causes both intestinal (diarrhea) and extraintestinal (UTI) infections, and this duality of disease is the unique feature that justifies the concept of heteropathogenicity for STEC O2:H6 (see the discussion in the revised manuscript, page 12, lines 5-8). We wish to put forward the concept of heteropathogens based on their phylogeny, virulence genotypes and phenotypes, and their pluripotential pathogenicity in intestinal and extraintestinal milieus. We therefore believe that these considerations, at least based on currently available data, fully support the statement in the Abstract that "STEC O2:H6 is….. the first such hybrid virulent *E. coli* identified".

3. Results, page 5, paragraph 1: additional information about the 13 O2:H6 isolates would be of interest: how many patients had bloody diarrhea? Did any patients develop hemolytic-uremic syndrome. Were other known enteric pathogens isolated?

Answer to 3: We have clarified this point and added the information that all patients had non-bloody diarrhea without progression to HUS and without evidence of any other enteric pathogens in the revised manuscript (page 5, lines 3-6).

4. Results, page 6, paragraph 2: it should be determined whether the O2:H6 strains contain type 1 pilus genes typical of UPEC and AIEC (Inflammatory Bowel Disease 2009;11: 737-745).

Answer to 4:

As suggested by referee #1, we have investigated the FimH sequences from the newly sequenced O2:H6 strains and also from UPEC and AIEC reference strains. Using the recently proposed FimH classification (Dreux et al., Plos Pathogens 2013), we were able to determine the FimH clade of the O2:H6 strains. Interestingly, they all grouped into the AIEC/UPEC FimH clade S70/N78. These results are now included in the Results section of the revised manuscript (page 7, lines 3-11) and in a new supplemental figure (Supporting Information Fig 1).

5. Results, page 8: is the urovirulence of O2:H6 specific? It would be of great interest to know whether O104:H4 and O83:H1 strains have comparable effects in murine models of cystitis and pylenophritis. It would also be relevant to determine whether O2:H6 isolates induce enteric disease in a relevant animal model (Nat Commun 2013; June10;4:1957).

Answer to 5: We agree with referee #1 that comparison of STEC O2:H6 with AIEC O83:H1 and E. coli O104:H4 strains for their potential to cause also urinary tract infections would be very interesting. However, performing such animal experiments largely exceeds the scope of our manuscript, which is focused on characterization of STEC O2:H6 as human pathogen rather than on comparison of the pathogenic potential of this organism with other enteric pathogenic E. coli. The latter topic requires detailed future studies.

We believe that the ability of STEC O26:H6 to cause diarrhea in humans (as indicated by the isolation of these strains as the only pathogens from patients with diarrhea) is the best proof of their ability to cause human enteric disease. Therefore, we believe that in our study we would not gain further information from an animal model, which is usually used either when the clinical effect of pathogens is not clear from the natural infection in humans or experiments in humans are not possible for ethical reasons. Finally, we think that the utility of the suggested animal model, which allows to determine the inflammatory response similar to that seen in Crohn´s disease, would be

hindered in case of STEC O2:H6 by the fact that the mice would be very likely killed by Shiga toxin produced by these organisms.

6. Discussion, page 11, paragraph 2: phylogenetic positioning between STEC and UPEC of serotypes other than O2:H6 (eg. O83:H1) must be acknowledged and then considered in some detail.

Answer to 6:

As we respond to comment 1 of referee #1, we have expanded on this topic by adding additional genomic analyses (see also Answer 1 to referee #2). The results of phylogenetic positioning of AIEC based on MLST, rMLST and whole genome sequencing are now shown in Figures 1 and 2 in the revised manuscript, where we included the AIEC reference strain LF82 of serotype O83:H1, and described in the Results section (page 5, lines 14-15 and 20-24, page 6, lines 1-2, and page 7, lines 3-4). Moreover, we have expanded the discussion by addressing the results of the phylogenetic positioning of AIEC (page 11, line 26 and page 12, lines 1-5).

Referee #2 (Comments on Novelty/Model System):

I basically believe that this is an interesting study on pathogen evolution, but I think they have to push the genomic analyses much further. Thus, for a paper in EMBO Molecular Medicine I think they need to make whole genome analyses of some ST141 isolates and make state of the art bioinformatic comparisons to their closest relatives among UPEC and HUSEC isolates.

Referee #2 (Remarks):

In this paper by Bielaszewska et al a set of diarrheangenic Shiga toxin producing E. coli (STEC) 02:H6 were shown to possess virulence attributes of STEC as well as uropathogenic E. coli. Phylogenetically these isolates were positioned between UPEC and HUSEC. The authors interpret their findings such that one pathogroup of E. coli may undergo phased metamorphosis from one pathogroup to another. Their findings that the core genome changes together with the virulence attributes is an argument for that virulence do not simply evolve by linear acquisition of virulence genes into a non-pathogenic background. However, the suggestion that the studied STEC ST141 isolates represent old transient hybrid pathogens that next over time may evolve into either UPEC or HUSEC is not fully convincing and difficult to distinguish from a model where these STEC isolates are genetic end products of more recent events involving one or more large horizontal gene transfers between UPEC and HUSEC isolates. To strenghten the idea put forward by the authors I would recommend them to fully sequence some of the ST141 isolates and do the same for UPEC and HUSEC isolates that are the most related to ST141. If ST141 represents a very old lineage where the core genome has co-evolved with the virulence attributes it would look different from a more recently evolved hybrid from already existing UPEC and HUSEC genomes.

Apart from this major comment I find this paper interesting and well written.

Answer to this comment:

As suggested, we performed whole genome sequencing in 5 STEC O2:H6 strains. We performed a gene-by-gene comparison of all 2827 genes that were present in all strains investigated including the five STEC O2:H6, UPEC and AIEC reference strains (as also suggested by referee #1) and HUS-associated STEC closely related to ST141, i.e. STEC O91:H21 (ST442) as well as prototypic HUS-associated STEC reference strains (STEC O157:H7, O26:H11; O111:H$^-$, O103:H2). We included these results in the revised manuscript as a new figure (Figure 2) and mention them in the Results section (page 5, lines 20-24, lpage 6, lines 1-2). Moreover, we discuss these results, also in the light

of the evolutionary position of AIEC (as requested by referee #1), in the revised manuscript in the Discussion section (page 10, lines 25-26 and page 11, lines 1-2; page 11, lines 15-17). Indeed, analysis of the core genome represented by 2827 genes present in all strains investigated corroborated our hypothesis of co-evolution of the genome of STEC O2:H6 with the virulence attributes rather than a recent evolution of this pathogen from already existing UPEC and HUSEC.

2nd Editorial Decision 04 November 2013

Thank you for the submission of your revised manuscript to EMBO Molecular Medicine. We have now received the enclosed reports from the referees that were asked to re-assess it. As you will see the reviewers are now supportive and I am pleased to inform you that we will be able to accept your manuscript pending the following final amendments:

- Please include Table 1 legend and Table 1 within the main manuscript file.

- We would need a short list of bullet points (up to 4-5) that summarize the key NEW findings. The bullet points should be sentences in a passive voice, designed to be complementary to the abstract and will be used online in our new platform coming January 2014.

I look forward to seeing a revised form of your manuscript as soon as possible.

***** Reviewer's comments *****

Referee #1:

The authors have provided a great deal of new data in this resubmission (and not simply cosmetic changes to the previous version). As a result of responding positively to the comments and suggestions provided previously by the two reviewers, the revised manuscript is much improved and more impactful than the previous submission.

Referee #2:

This revised version of the manuscript has further strengthened the paper. They have adequately responded to my main comment and performed the requested whole genome sequencing. I have no further comments.
