# Supplementary material for: Heteropathogenic virulence and phylogeny reveal phased pathogenic metamorphosis in Escherichia coli O2:H6
Source: EMBO Mol Med. 2014 Jan 10;6(3):347–57. doi: 10.1002/emmm.201303133 (PMC3958309; doi:10.1002/emmm.201303133)
Supplement: Supplementary file 2 [file emmm0006-0347-sd2.pdf]

Supporting Information Figures

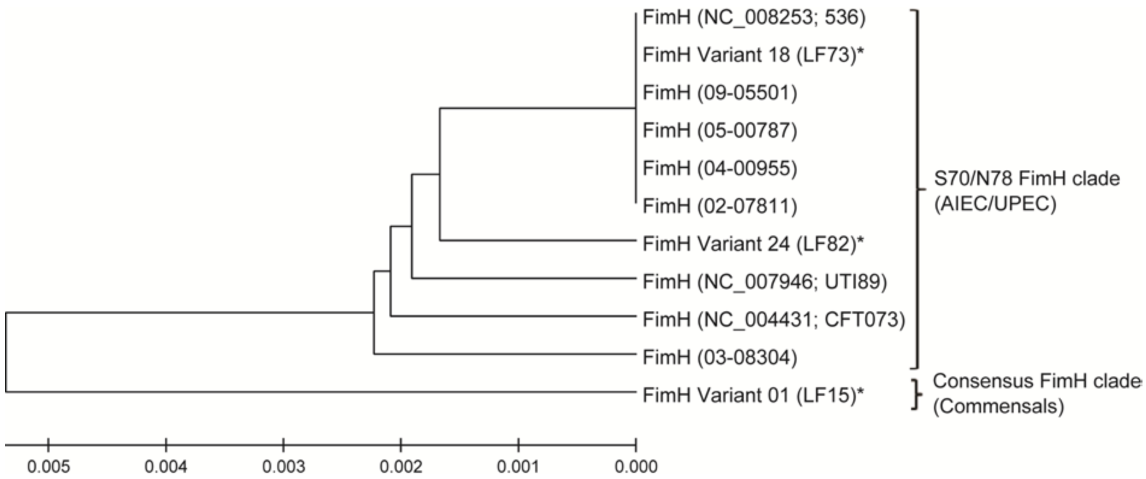

Supporting Information Fig 1. UPGMA-tree of FimH protein sequences of STEC O2:H6 and AIEC and UPEC reference strains. The nomenclature of the FimH variants marked with an * and FimH clades is based on Dreux et al. (Dreux et al, 2013). In parenthesis, the GenBank accession numbers, if applicable, and the isolate names of the respective protein sequence are given.
