# Supplementary material for: Heteropathogenic virulence and phylogeny reveal phased pathogenic metamorphosis in Escherichia coli O2:H6
Source: EMBO Mol Med. 2014 Jan 10;6(3):347–57. doi: 10.1002/emmm.201303133 (PMC3958309; doi:10.1002/emmm.201303133)
Supplement: Supplementary file 3 [file emmm0006-0347-sd3.pdf]

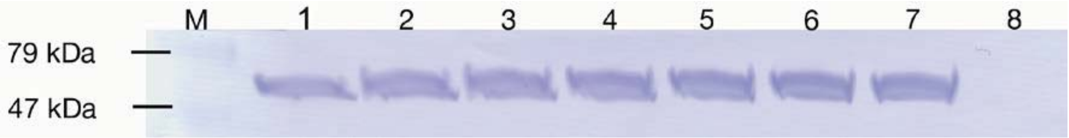

Supporting Information Fig 2. Expression of Saa by STEC O2:H6. Bacterial lysates were separated by sodium dodecyl sulfate polyacrylamide gel electrophoresis (SDS-PAGE), and proteins transferred onto a membrane were probed with an anti-Saa antibody and alkaline phosphatase-conjugated goat anti-mouse IgG. In lanes 1 to 7 following *saa*-positive STEC O2:H6 strains are shown: Lane 1, 00-03365; lane 2, 02-07811; lane 3, 03-03095; lane 4, 04-03909; lane 5, 05-00787; lane 6, 05-06739; and lane 7, 09-05501; lane 8 contains *saa*-negative STEC O2:H27 strain 05-07247. M, protein size marker (peqGOLD Prestained Protein-Marker III; Peqlab Biotechnologie, Erlangen, Germany). Signals in lanes 1 to 7 are representative of those displayed by all 13 *saa*-positive STEC O2:H6.
