# Supplementary material for: Heteropathogenic virulence and phylogeny reveal phased pathogenic metamorphosis in Escherichia coli O2:H6
Source: EMBO Mol Med. 2014 Jan 10;6(3):347–57. doi: 10.1002/emmm.201303133 (PMC3958309; doi:10.1002/emmm.201303133)
Supplement: Supplementary file 4 [file emmm0006-0347-sd4.pdf]

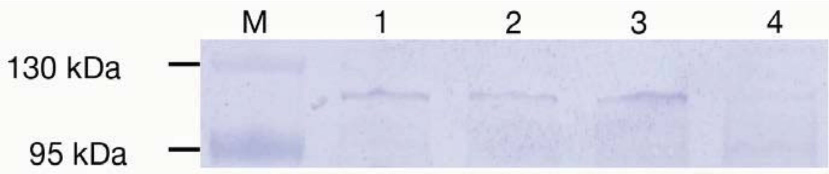

Supporting Information Fig 3. Expression of CNF1 by STEC O2:H6. Bacterial lysates were separated by SDS-PAGE, and proteins transferred onto a membrane were subjected to immunoblot with an anti-CNF1 antibody and alkaline phosphatase-conjugated goat anti-mouse IgG. In lanes 1 to 4 following strains are shown: Lane 1, *cnf1*-positive UPEC strain J96 (control); 2, *cnf1*-positive STEC O2:H6 strain 00-03365; 3, *cnf1*-positive STEC O2:H6 strain 04-03909; 4, *cnf1*-negative STEC O2:H6 strain 05-00787. M, protein size marker (peqGOLD Prestained Protein-Marker IV; Peqlab Biotechnologie). Similar reactions as shown in lanes 2 and 3 were produced by the other eight *cnf1*-positive STEC O2:H6 isolates.
