# Supplementary material for: Heteropathogenic virulence and phylogeny reveal phased pathogenic metamorphosis in Escherichia coli O2:H6
Source: EMBO Mol Med. 2014 Jan 10;6(3):347–57. doi: 10.1002/emmm.201303133 (PMC3958309; doi:10.1002/emmm.201303133)
Supplement: Supplementary file 6 [file emmm0006-0347-sd6.pdf]

Supporting Information Table 1. *Escherichia coli* strains used in this study[a]

Strain designation	Serotype[b]	Pathogroup[c]	ST[d]	Phylo-group[e]	*stx*[f]	Clinical diagnosis or source	Reference or accession number
00-03365	O2:H6	STEC	141	B2	stx_{2b}^{*}	Diarrhea	This study
02-07811[g]	O2:H6	STEC	141	B2	stx_{2b}	Diarrhea	This study
03-00945	O2:H6	STEC	141	B2	stx_{2b}	Diarrhea	This study
03-03095	O2:H6	STEC	141	B2	stx_{2b}^{*}	Diarrhea	This study
03-08304[g,h]	O2:H6	STEC	141	B2	stx_{2b}^{*}	Diarrhea	This study
03-08411	O2:H6	STEC	141	B2	stx_{2b}	Diarrhea	This study
04-00955[g]	O2:H6	STEC	141	B2	stx_{2b}	Diarrhea	This study
04-01674	O2:H6	STEC	141	B2	stx_{2b}	Diarrhea	This study
04-03909	O2:H6	STEC	141	B2	stx_{2b}^{*}	Diarrhea	This study
05-00787[g]	O2:H6	STEC	141	B2	stx_{2b}^{*}	Diarrhea	This study
05-00826	O2:H6	STEC	141	B2	stx_{2b}	Diarrhea	This study
05-06739	O2:H6	STEC	141	B2	stx_{2b}	Diarrhea	This study
09-05501[g]	O2:H6	STEC	141	B2	stx_{2b}^{*}	Diarrhea	This study
04-06991	O2:H4	STEC	405	D	n.k.	Asymptomatic carrier	This study
05-07247[g]	O2:H27	STEC	10	A	stx_{2a}	Water	This study
3750/1	O2:H29	STEC	515	B1	stx_{2c}	Cattle feces	(Tasara et al, 2008)
2348/69[h]	O127:H6	EPEC	15	B2	-	Diarrhea	NC_011601
E24377A[h]	O139:H28	ETEC	1132	B1	-	Diarrhea	NC_009801
53638[h]	O144:H^{-}	EIEC	6	n.a.	-	Diarrhea	NZ_AAKB02000000
042[h]	O44:H18	EAEC	414	D	-	Diarrhea	NC_017626
55989[h]	O104:H4	EAEC	678	B1	-	Diarrhea	NC_011748
536[h]	O6:K15:H31	UPEC	127	B2	-	Pyelonephritis	NC_008253
CFT073[h]	O6:K21:H1	UPEC	73	B2	-	Pyelonephritis	NC_004431
J96	O4:K6:H5	UPEC	12	B2	-	Pyelonephritis	(Blum et al, 1995)
UTI89[h]	O18:K1:H7	UPEC	95	B2	-	Cystitis	NC_007946
S88[h]	O45:K1:H7	MNEC	95	B2	-	Neonatal meningitis	NC_011742
IHE3034[h]	O18:K1:H7	MNEC	95	B2	-	Neonatal meningitis	NC_017628
LF82[h]	O83:H1	AIEC	135	B2	-	Crohn's disease	NC_011993
NRG 857C[h]	O83:H1	AIEC	135	B2	-	Crohn's disease	NC_017634
MG1655[h]	OR:H48	non-pathogenic	10	A	-	Commensal	NC_000913

[a] For characteristics of strains of the HUSEC collection (Mellmann et al, 2008) see http://www.ehec.org.

[b] H^{-}, nonmotile strain; OR, rough (autoagglutinable) strain.

[c] STEC, Shiga toxin-producing, EPEC, enteropathogenic, ETEC, enterotoxigenic, EIEC, enteroinvasive, EAEC, enteroaggregative, UPEC, uropathogenic, MNEC, meningitis-associated, AIEC, adherent-invasive *E. coli.*

[d] ST (sequence type) determined by multilocus sequence typing.

[e] The phylogroup was determined by triplex PCR (Clermont et al, 2000); n.a., not available.

[f] *stx* gene subtypes were determined by PCR and sequencing. stx_2 (GenBank accession no. GU126552) subtyped as stx_{2b} (Scheutz et al, 2012) is present in all *stx*-sequenced O2:H6 strains (*); n.k., *stx* subtype is not known because *stx* was lost before subtyping; -, *stx* absent.

[g] Strains subjected to whole genome shotgun sequencing for deeper phylogenetic analysis.

[h] Strains included in the phylogenetic tree based on MLST and rMLST sequence typing (Fig 1) in addition to the 42 strains of the HUSEC collection (Mellmann et al, 2008).
