# Supplementary material for: Heteropathogenic virulence and phylogeny reveal phased pathogenic metamorphosis in Escherichia coli O2:H6
Source: EMBO Mol Med. 2014 Jan 10;6(3):347–57. doi: 10.1002/emmm.201303133 (PMC3958309; doi:10.1002/emmm.201303133)
Supplement: Supplementary file 7 [file emmm0006-0347-sd7.pdf]

Supporting Information Table 3. Presence of virulence factors of other intestinal *E. coli* pathogroups in STEC O2:H6

Pathogroup[a]	Virulence factor	Presence in STEC O2:H6[b]
EPEC	Locus of enterocyte effacement (LEE)[c]	0/13
ETEC	Heat-labile enterotoxin (LT)	0/13
	Heat-stable enterotoxin (STI)	0/13
EIEC	Invasive plasmid (pInv)[d]	0/13
	Shigella enterotoxin 2	0/13
EAEC	EAEC virulence plasmid[e]	0/13
	EAEC heat-stable enterotoxin 1	0/13
	Shigella enterotoxin 1	0/13
	Autotransporter Pet (plasmid-encoded toxin)	0/13
	Autotransporter Pic (protein involved in intestinal colonization)	0/13

[a] EPEC, enteropathogenic, ETEC, enterotoxigenic, EIEC, enteroinvasive, EAEC, enteroaggregative *E. coli*.

[b] Number of strains positive for the locus/number of tested.

[c] Tested by PCRs targeting *eae* (encoding intimin), *escV* (component of the type III secretion system), and *espF*, *map*, and *espG* (encoding secreted effector proteins).

[d] Tested by PCR targeting the region of pInv used as an EIEC diagnostic probe (*ial*).

[e] Tested by PCR targeting the region of the EAEC virulence plasmid used as an EAEC diagnostic probe (pCVD432) and corresponding to *aatA* locus.
