# Supplementary material for: Heteropathogenic virulence and phylogeny reveal phased pathogenic metamorphosis in Escherichia coli O2:H6
Source: EMBO Mol Med. 2014 Jan 10;6(3):347–57. doi: 10.1002/emmm.201303133 (PMC3958309; doi:10.1002/emmm.201303133)
Supplement: Supplementary file 8 [file emmm0006-0347-sd8.pdf]

Supporting Information Table 4. PCR primers and conditions used to analyze the presence of the *cdiAB* cluster and *clb* island

Primer[a]	Sequence (5´- 3´)	Target	PCR conditions[b]			Amplicon (bp)	Position of primer[c]
			Denaturing	Annealing	Extension		
CdiB-100-f	GTACGCTATTTTTCCCTCCTC	*cdiB 5´ end*	94°C, 30 s	57°C, 60 s	72°C, 60s	543	2596-2616
CdiB-622-r	CTCAATATCCCGCAGGTTCAG						3138-3118
CdiB-1161-f	CAGCAGCCGTAAACTCACTTC	*cdiB 3´ end*	94°C, 30 s	57°C, 60 s	72°C, 60s	384	3657-3677
CdiB-1524-r	TATGGCAGGGAGAACAGAGAC						4040-4020
CdiB-f	GAGACCTGCCCGTAAATC	*cdiB-cdiA*	94°C, 30 s	56°C, 60 s	72°C, 90s	819	3794-3811
CdiA-r	CGTTGATGATACCCTTCG						4612-4595
CdiA-281-f	TGATACAGAATAACCCGAACC	*cdiA 5´ end*	94°C, 30 s	57°C, 60 s	72°C, 60s	655	4556-4576
CdiA-915-r	TTGTTATCCCCTGAAAGTGAG						5210-5190
CdiA-2746-f	AGCACAGCAGGCAGCAAAGTC	*cdiA* middle	94°C, 30 s	58°C, 60 s	72°C, 60s	494	7021-7041
CdiA-3219-r	GAGAGGATTTTGCCGTCGTTC						7514-7494
P-pks1	TTCCCACGGTCTGTATCTGC	*clb* island *5´ end*	94°C, 30s	56°C, 60s	72°C, 60s	355	534-553
P-pks2	AAGCTCGGTAACTGGTAGATG						888-868
P-pks3	CGCTTCATCAACACGCTTTA	*clb* island middle	94°C, 30s	58°C, 60s	72°C, 60s	290	29904-29923
P-pks4	CCATCGCCTATCACCTCAAC						30193-30174
P-pks7	TCGCATTAGCCTCTCCACTC	*clb* island *3´ end*	94°C, 30s	52°C, 60s	72°C, 60s	232	52672-52691
P-pks8	TGCCGGATGATCTCTAAATG						52903-52884

[a] All primers except for P-pks3 (Nougayrède et al, 2006) were designed in this study.

[b] All PCRs included 30 cycles as given in the table preceded by initial denaturation (94°C, 5 min) and followed by final extension (72°C, 5 min).

[c] Positions of the primers for the detection of the *cdiAB* cluster are in the *cdiAB* sequence of *E. coli* strain EC93 (GenBank accession no. DQ100454). Positions of the primers for the detection of the *clb* island are in the sequence of the colibactin polyketide biosynthesis gene cluster of *E. coli* strain IHE3034 (GenBank accession no. AM229678).
