# Supplementary material for: Heteropathogenic virulence and phylogeny reveal phased pathogenic metamorphosis in Escherichia coli O2:H6
Source: EMBO Mol Med. 2014 Jan 10;6(3):347–57. doi: 10.1002/emmm.201303133 (PMC3958309; doi:10.1002/emmm.201303133)
Supplement: Supplementary file 9 [file emmm0006-0347-sd9.pdf]

Supporting Information – Table of Contents

Files in the Supporting Information

Supporting Information Tables

Supporting Information Table 1. *Escherichia coli* strains used in this study.

Supporting Information Table 2. rMLST allelic profiles of the different *E. coli* pathogroups in accordance to the rMLST database (Jolley et al, 2012).

Supporting Information Table 3. Presence of virulence factors of other intestinal *E. coli* pathogroups in STEC O2:H6.

Supporting Information Table 4. PCR primers and conditions used to analyze the presence of the *cdiAB* cluster and *clb* island.
